# Efficacy and safety of double balloon catheter and dinoprostone for labor induction in multipara at term

**DOI:** 10.1007/s00404-022-06891-9

**Published:** 2023-02-20

**Authors:** Lu Yuan, Jing Peng, Lijun Yang, Yun Zhao

**Affiliations:** Department of Obstetrics, Maternal and Child Health Hospital of Hubei Province, Tongji Medical College, Huazhong University of Science and Technology, No. 745, Wuluo Road, Hongshan District, Wuhan, 430070 China

**Keywords:** Multipara, Double balloon catheter, Dinoprostone, Induction of labor, Vaginal delivery

## Abstract

**Purpose:**

The aim of this study was to comparatively assess the efficacy and safety of double balloon catheter (DBC) and dinoprostone as labor-inducing agents just for multipara at term.

**Methods:**

A retrospective cohort study was conducted among multipara at term with a Bishop score < 6 who needed planned labor induction from January 1, 2020, to December 30, 2020 in Maternal and Child Health Hospital of Hubei province, Tongji Medical College, Huazhong University of Science and Technology. They were divided into DBC group and dinoprostone group, respectively. Baseline maternal data, maternal and neonatal outcomes were recorded for statistical analysis. Total vaginal delivery rate, rate of vaginal delivery within 24 h, rate of uterine hyperstimulation combined with abnormal fetal heart rate(FHR) were regarded as the primary outcome variables. The difference between groups was considered statistically significant when *p* value < 0.05.

**Results:**

A total of 202 multiparas was included for analysis (95 women in DBC group vs 107 women in dinoprostone group). There were no significant differences in total vaginal delivery rate and rate of vaginal delivery within 24 h between groups. Uterine hyperstimulation combined with abnormal FHR occurred exclusively in dinoprostone group.

**Conclusion:**

DBC and dinoprostone seem to be equally effective, while, DBC seems to be safer than dinoprostone.

## What does this study add to the clinical work

**Table:** 

This is the first time to compare the efficacy and safety of double balloon catheter and dinoprostone for labor induction just in multipara at term, providing more data for clinical practice guidelines.	

## Introduction

Induction of labor (IOL) is an obstetrical procedure that has been increasingly used in recent years. The proportion of people experiencing IOL increased by nearly 10% from 2007 to 2017, with IOL rate exceeding 25.5% (1 in 4) in 2017 [1]. With the implementation of two-child policy, the rate of labor induction at term has been increased to more than 30% in 2013 in China [2]. Following the publication of the ARRIVE trial [3] and the implementation of three-child policy, the IOL rate is expected to rise in the future in China.

For women with an unfavorable cervix (Bishop score < 6) [4–6], the additional step of cervical ripening is required during induction of labor. There are mechanical and pharmacologic methods of cervical ripening. Approved by the United States Food and Drug Administration (FDA) in 2013, the Cook Cervical Ripening Balloon (Cook Inc; Bloomington, IN) can lead to cervical ripening by either direct mechanical dilation of the cervix or stimulation of prostaglandin released from the amnion, chorion, and decidua [7, 8]. Dinoprostone is chemically identical to endogenous prostaglandin E2 (PGE2), which has been approved by the FDA for cervical ripening and has been widely used in several countries throughout the world. There have been many studies comparatively studying the effectiveness and safety of the two labor induction methods [9–13]. It is well known that the history of vaginal delivery itself is of vital importance for the success of induction, but the vast majority of studies have focused on primipara or have not fully distinguished between primipara and multipara. However, the optimal method for labor induction for multipara at term with an unfavorable cervix remains unknown.

In this retrospective cohort study conducted in our birth centre from January 1, 2020, to December 30, 2020, the efficacy and safety of DBC and dinoprostone as labor-inducing agents for multipara were comparatively analyzed.

## Materials and methods

### Ethical approval and patient consent

The study protocol was approved by the Ethics Committee of Maternal and Child Health Hospital of Hubei Province, Tongji Medical College, Huazhong University of Science and Technology [(2019) IEC (XM008)]. All included women signed written informed consent for therapeutic procedures and for the publication of those reports.

### Selection of patients and study design

**Fig. 1:**
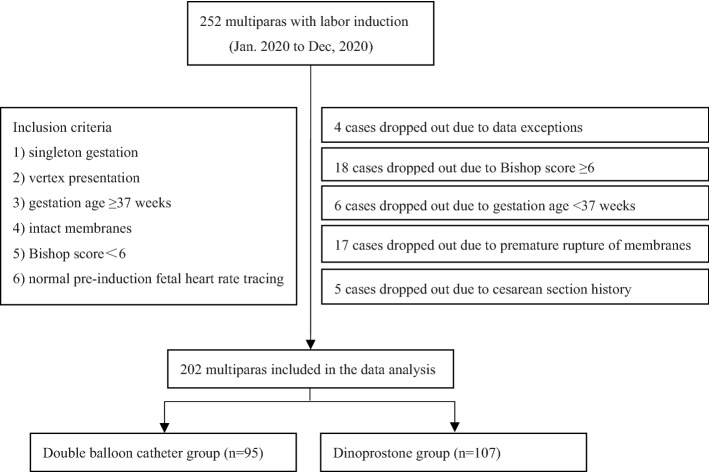
Flow Diagram

The flowchart of the experimental design is shown in Fig. 1. In this retrospective cohort study, a total of multiparas aged 18–50 years with gestational ages ≥ 37 weeks, history of previous vaginal delivery, singleton gestation, vertex presentation, intact membranes, normal preinduction fetal heart rate tracing, Bishop score < 6, fetal weight of less than 4500 g with a ultrasound or clinical estimated were included. The exclusion criteria were pregnant women aged less than 18 years or older than 50 years, primipara, previous cesarean section. During the observation period at our birth centre from January 1st, 2020 to December 30th, 2020, a total of 252 multiparas were enrolled in this study. Among them, 4 cases with missing data, 18 cases with Bishop scores ≥ 6, 6 cases with gestation age < 37 weeks by ultrasound, 17 cases with premature rupture of membranes, and 5 cases with prior cesarean section history were excluded, and the remaining 202 cases were included in our study. Based on the methods of IOD, the 202 cases were divided into two groups: DBC group (*n* = 95) and dinoprostone group (*n* = 107).

The multiparas in DBC group were treated with DBC (Cervical Ripening Balloon; Cook OB/GYN, Spencer, IN, USA) for labor induction. The DBC involved 2 balloons (uterine and vaginal balloons). First, the uterine balloon (red piston, marked with “U”) was inserted into uterine cavity by long oval forceps under direct visualization and 40 mL of normal saline solution was injected in. Then the vaginal balloon (green piston, marked with “V”) was pulled out of the cervical orifice slightly, and 40 mL of normal saline solution was injected in. When they were correctly situated on either end of the cervix, the fluid amount in both balloons was alternatively increased by 20 mL each time until each balloon reached 80 mL. The external end of the device was taped to the patient’s leg without tension after ensuring that the balloons were positioned correctly. When symptoms of sweating or flustering were unbearable, then 10–20 mL of normal saline was withdrawn from both balloons until the patient could tolerate the DBC. The DBC should be removed immediately upon the occurrence of any of the following events, including spontaneous labor, expulsion, spontaneous ruptured membranes, or unexplained vaginal bleeding. If those events do not happen, the DBC device will be removed after holding for 12 h [14].

The multiparas in dinoprostone group were induced with a slow-release vaginal insert containing 10 mg of dinoprostone (prostaglandins PGE2 Propess^®^, Ferring SAS, Gentilly, France). The slow-release vaginal insert was stored in a freezer at a temperature between − 20 and − 10 °C. Dinoprostone should be removed in case of spontaneous exit, labor, being placed in the vagina for over 24 h.

After the removal or self-expulsion of the IOL agents, patients underwent a vaginal examination and then transferred to the delivery ward for spontaneous labor or augmentation by oxytocin infusion and/or artificial rupture of membranes with a 60-min interval if uterine contractions were not adequate. Epidural analgesia was provided under maternal request after uterine orifice dilation was more than or equal to 1 cm. Continuous monitoring of uterine activity and fetal heart rate was performed during active labor.

### Observation indicators

Information including maternal age, maternal body mass index (BMI) before pregnancy and at time of IOL, gestational age at time of delivery, gravidity, parity, abortion history, vaginal delivery history, indication for IOL, initial Bishop score, labor and perinatal period were collected and recorded in a form specially designed for this trial. To compare the efficacy and safety of DBC and dinoprostone for labor induction, total vaginal delivery rate, rate of vaginal delivery within 24 h, rate of uterine hyperstimulation combined with abnormal fetal heart rate and rate of fetal distress were regarded as the primary outcome variables. Secondary outcome variables include indications for cesarean section, insertion to active labor, length of first stage of labor, length of second stage of labor, length of third stage of labor, length of total labor, oxytocin augmentation, artificial rupture of membrane, postpartum hemorrhage, meconium staining of amniotic fluid, precipitate labor, episiotomy, perineal laceration, intrapartum fever, neuraxial analgesia. Neonatal outcomes include newborn weight, neonatal asphyxia, neonatal intensive care unit (NICU) admission. Cesarean section on maternal request is defined as the cesarean section based solely on maternal request without any maternal or fetal medical indications. Insertion to active labor is defined as the time from placing the DBC or applying dinoprostone to the start of regular contractions. Uterine hyperstimulation is defined as contractions more than 5 in 10 min for more than 20 min or contractions lasting more than 2 min in duration [15]. Fetal distress is defined as the symptoms that endanger the health and life of the fetus in utero due to acute or chronic hypoxia. Precipitate labor is defined as labor not exceeding 3 h. Failed induction is defined as no active labor within 48 h of induced labor. Neonatal asphyxia is defined as having an Apgar score ≤ 7 at 1 min. Adverse neonatal outcome variables include neonatal asphyxia, neonatal intensive care unit admission.

### Statistical methods

All analyses were conducted using the Statistical Package of Social Sciences software (SPSS Version 26.0 Inc., Chicago, IL, USA). Continuous variables were presented as means ± standard deviation and categorical variables were presented as frequency and percentage (%). Student’s *t* test was performed to compare the variables in a Gaussian distribution. The chi-square test or Fisher’s exact test were used to evaluate the categorical variables. The Mann–Whitney test was used to evaluate the difference in a non-Gaussian distribution between two groups. The difference was considered statistically significant when *p* < 0.05.

## Results

As shown in Table 1, baseline characteristics of multiparas with labor induction in DBC group and dinoprostone group were comparatively analyzed. There were no significant differences in maternal age, BMI at delivery, gravidity, parity, abortion history, vaginal delivery history, indication for IOL, initial Bishop score between the two groups (*p* > 0.05).

**Table 1:** Baseline characteristics of multiparas between in the DBC and Dinoprostone

Characteristics	DBC	Dinoprostone	*p* value
	(*n* = 95)	(*n* = 107)	
Age (y,$\overline{X }\pm s$)	32.3 ± 4.1	32.2 ± 2.9	0.839
≥ 35 years (*n*, %)	27 (28.4)	23 (21.5)	0.255
BMI before pregnancy (kg/m^2^$,\overline{ X }\pm s$)	21.3 ± 2.4	21.9 ± 2.7	0.075
BMI at time of IOL (kg/m^2^$,\overline{ X }\pm s$)	26.5 ± 3.0	27.1 ± 2.7	0.086
Gestational age at time of delivery (w, $\overline{\mathrm{X}}\pm \mathrm{s }$)	39.8 ± 1.0	39.8 ± 0.9	0.695
Gravidity(min–max)	2–5	2–9	0.386
Parity(min–max)	1.0 ± 0.1	1.0 ± 0.2	0.497
Abortion history (*n*, %)			
None	39 (41.1)	52 (48.6)	0.282
Once	28 (29.5)	33 (30.8)	
Twice	17 (17.9)	12 (11.2)	
Three times	11 (11.6)	8 (7.5)	
≥ Four times	0 (0)	2 (1.9)	
Vaginal delivery history (*n*, %)			
Once	93 (97.9)	103 (96.3)	0.495
Twice	2 (2.1)	4 (3.7)	
Indication for IOL (*n*, %)			
Social/elective	16 (16.8)	26 (24.3)	0.259
Delayed gestation	15 (15.8)	9 (8.4)	
Hypertensive disorder	7 (7.4)	7 (6.5)	
Gestational diabetes	40 (42.1)	52 (48.6)	
Suspected oligohydramnios	17 (17.9)	13 (12.2)	
Neuraxial labor analgesia (*n*, %)	53 (55.8)	51 (47.7)	0.249
Initial bishop score ($\overline{X }\pm s$)	4.2 ± 0.6	4.4 ± 0.6	0.080

Student’s *t* test, chi-square test and Mann–Whitney test were used

*p* < 0.05 was considered significant

*DBC* double balloon catheter, *BMI* body mass index, *IOL* induction of labor

Overall, natural delivery rate, cesarean section rate and forceps delivery rate were 89.1% (180/202), 9.9% (20/202) and 1.0% (2/202), respectively. The total vaginal delivery rate was 93.7% in DBC group and 86.9% in dinoprostone group, with no significant difference between the two groups (*p* > 0.05), as shown in Table 2. The vaginal delivery (including forceps) rates within 24 h, 36 h, 48 h, > 48 h were 75.8%, 88.4%, 92.6%, 93.7% in DBC group, and 71.0%, 82.2%, 83.2%, 86.9% in dinoprostone group, and there was no statistical differences between in the two groups (*p* > 0.05), as shown in Table 2.

**Table 2:** Comparison of vaginal birth rate within different parturition time between in DBC and Dinoprostone

	24 h		36 h		48 h		> 48 h	
Delivery mode	DBC (*n* = 95)	Dinoprostone (*n* = 107)	DBC (*n* = 95)	Dinoprostone (*n* = 107)	DBC (*n* = 95)	Dinoprostone (*n* = 107)	DBC (*n* = 95)	Dinoprostone (*n* = 107)
Vaginal delivery (*n*, %)	72 (75.8)	76 (71.0)	84 (88.4)	88 (82.2)	88 (92.6)	89 (83.2)	89 (93.7)	93 (86.9)
Cesarean delivery (*n*, %)	6 (6.3)	8 (7.5)	6 (6.3)	9 (8.4)	6 (6.3)	12 (11.2)	6 (6.3)	14 (13.1)
*p* value	0.679		0.511		0.195		0.108	

Chi-square test was used

*p* < 0.05 was considered significant

*DBC* double balloon catheter

Further, the indications for cesarean section in DBC group included fetal distress (*n* = 4), antepartum hemorrhage (*n* = 1), maternal request cesarean section (*n* = 1), and those in dinoprostone included fetal distress (*n* = 11), failed induction (*n* = 2), cesarean section on maternal request (*n* = 1).

The main maternal outcome variables of DBC group and in dinoprostone group were comparatively analyzed (Table 3). The rate of uterine hyperstimulation combined with abnormal FHR was higher in the dinoprostone group than in the DBC group (5.6% vs. 0%, *p* = 0.019), and the rates of meconium staining of amniotic fluid and intrapartum fever in the dinoprostone group were higher than in the DBC group (19.6% vs. 9.5%, *p* = 0.043 and 19.6% vs. 8.4%, *p* = 0.023). The rates of oxytocin augmentation and artificial rupture of membrane were higher in the DBC group than in the dinoprostone group (77.9% vs. 19.6%, *p* < 0.001 and 63.2% vs. 10.3%, *p* < 0.001). Moreover, there were no significant difference in the rates of precipitate delivery, fetal distress, neuraxial analgesia, postpartum hemorrhage, episiotomy, perineal laceration between the two groups (*p* > 0.05). The median time of insertion to active labor in the DBC group was 992.3 m, which was longer than that in the dinoprostone group (753.2 m, *p* < 0.001), but there were no significant difference in the median time of first stage of labor, second stage of labor, third stage of labor and the total labor between the two groups (*p* > 0.05).

**Table 3:** Maternal outcomes between DBC and Dinoprostone

Outcomes	DBC	Dinoprostone	*p* value
	(*n* = 95)	(*n* = 107)	
Insertion to active labor (m, $\overline{ X }\pm s$)	992.3 ± 430.9	753.2 ± 766.8	< 0.001
Length of first stage of labor (m, $\overline{ X }\pm s$)	253.3 ± 109.8	283.1 ± 133.8	0.102
Length of second stage of labor (m, $\overline{\mathrm{X}}\pm \mathrm{s }$)	14.7 ± 12.0	15.0 ± 13.2	0.872
Length of third stage of labor (m, $\overline{ X }\pm s$)	9.8 ± 7.7	9.0 ± 6.7	0.459
Length of total labor (m, $\overline{ X }\pm s$)	277.8 ± 112.5	307.1 ± 138.7	0.121
Neuraxial analgesia (*n*, %)	53 (55.8)	51 (47.7)	0.249
Oxytocin augmentation (*n*, %)	74 (77.9)	21 (19.6)	< 0.001
Artificial rupture of membrane (*n*, %)	60 (63.2)	11 (10.3)	< 0.001
Postpartum hemorrhage (≥ 500 ml) (*n*, %)	8 (8.4)	6 (5.6)	0.432
Uterine hyperstimulation with FHR changs (*n*, %)	0 (0)	6 (5.6)	0.019
Amniotic fluid fecal staining (*n*, %)	9 (9.5)	21 (19.6)	0.043
Prenatal fever (*n*, %)	8 (8.4)	21 (19.6)	0.023
Precipitate labour (*n*, %)	8 (8.4)	13 (12.2)	0.386
Episiotomy (*n*, %)	2 (2.1)	6 (5.6)	0.203
Perineal laceration (*n*, %)	3 (3.2)	3 (2.8)	0.882

Student’s *t* test, Chi-square test or Fisher’s exact test and Mann–Whitney test were used

*p* < 0.05 was considered significant

*DBC* double balloon catheter

Table 4 shows the main outcomes of newborns**.** Neonatal outcomes were similar in both study groups. The average birth weight in the DBC group and the dinoprostone group was 3399.1 ± 383.7 g and 3480.8 ± 381.0 g (*p* = 0.131). There were two newborns having an Apgar ≤ 7 at 1 min in the dinoprostone group, but one newborn in the DBC group. There were two newborns staying in neonatal intensive care unit for each groups.

**Table 4:** Fetal and neonatal outcomes between DBC and Dinoprostone

Outcomes	DBC	Dinoprostone	*p* value
	(*n* = 95)	(*n* = 107)	
Fetal distress (*n*, %)	4 (4.2)	11 (10.3)	0.101
Birthweight (g, $\overline{X }\pm s$))	3399.1 ± 383.7	3480.8 ± 381.0	0.131
Adverse neonatal outcome (*n*, %)	2 (2.1)	2 (1.9)	0.904
Neonatal asphyxia (*n*, %)	1 (1.1)	2 (1.9)	0.632
NICU (*n*, %)	2 (2.1)	2 (1.9)	0.904

Student’s *t* test, Chi-square test or Fisher’s exact test were used

*p* < 0.05 was considered significant

*DBC* double balloon catheter

## Discussion

Nowadays, double balloon catheter (DBC) and dinoprostone as two useful methods for induction of labor in pregnant women at term have been widely used in clinical practice [16–18]. In our trial, we compared the efficacy and safety of DBC and dinoprostone as labor-inducing agents just in multipara at term. Our findings indicate that DBC and Dinoprostone seem to be equally effective since there were no differences in rate of vaginal delivery within 24 h, total vaginal delivery rate and cesarean section rate under the two different methods. However, DBC seems to have a more higher safety than dinoprostone as it led to lower rates of uterine hyperstimulation combined with abnormal FHR, amniotic fluid fecal staining, renatal fever, and there was a downward trend of fetal distress in DBC group compared with dinoprostone group.

The rate of vaginal delivery within 24 h was recommended by members of guidelines development groups in WHO (World Health Organization) and NICE (National Institute for Health and Clinical Excellence) as the most clinically relevant indication to evaluate the effectiveness of labor induction methods. Moreover, vaginal delivery rate and cesarean section rate have also been used as important indicators to evaluate the effectiveness of labor induction methods. Several studies have compared the efficacy of balloon catheters and dinoprostone, and conflicting results were obtained. A Cochrane analysis [19] found that there may be little or *n* significant difference in the rate of vaginal delivery within 24 h ((RR 1.01, 95% CI 0.82–1.26) and rate of cesarean section (RR 1.00, 95% CI 0.92–1.09) when using balloon and vaginal PGE2 as labor-inducing agents, which is consistent with the results of another analysis [20]. Du et al. [21] reported that the overall vaginal delivery rate in women treated with double balloon catheter was similar with that of dinoprostone (71.6% vs. 62.8%; *p* > 0.05), but more women were vaginally delivered within 24 h in dinoprostone group (52.26% vs. 37.62%, *p* = 0.0079)**.** Suffecool et al. [22] reported that more women were vaginally delivered within 24 h in double balloon catheter group (87.1% vs. 47.4%, *p* = 0.002) than in dinoprostone group. In our trial, there were no significant differences in rate of total vaginal delivery rate, rate of vaginal delivery within 24 h and rate of cesarean section between DBC group and dinoprostone group, although the time from start of induction to active labor was longer in DBC group than in dinoprostone group, and these results are similar to those reported in the recent meta-analysis mentioned above [19, 20].

For a method to induce labor, safety seems to be a more important index than efficacy. As an exogenous PGE2, dinoprostone can not only stimulate cervical remodeling, but also initiate uterine contractions via stimulating endogenous prostaglandin F2α production or sensitizing the myometrium to the effects of endogenous or exogenous oxytocin [18]. The most significant adverse event associated with dinoprostone is uterine hyperstimulation. Wing et al. [23] found that the rate of uterine hyperstimulation combined with FHR accounted for 4.0% in the dinoprostone group, while Rugarn et al. [24] reported this rate was 1.2%. DBC was used to induce labor by mechanically dilating the cervix and stimulating the release of prostaglandins from the amniotic membrane, chorionic membrane and decidua to ripen the cervix. In the Cochrane analysis [19]**,** a balloon catheter probably reduces the rate of uterine hyperstimulation combined with FHR (RR 0.35, 95% CI 0.18–0.67), rate of serious neonatal morbidity or perinatal death (RR 0.48, 95% CI 0.25–0.93) and may slightly reduce the rate of aneonatal intensive care unit (NICU) admission (RR 0.82, 95% CI 0.65–1.04). A recent trial conducted by Grace et al. published in 2021 [15] reported that the rates of uterine hyperstimulation and fetal distress were lower in DBC group than vaginal prostaglandin group in the first 12 h. However, a multicentre randomized controlled trial in Australian [10] showed that there were no statistically significant differences in the primary outcome variables (18.6% vs. 25.8%; RR 0.77, 95% CI 0.51–1.02; *p* = 0.070) or in the rate of meconium stained liquor (12.6% vs. 11.2%, *p* = 0.647) between DBC group and dinoprostone group, but uterine hyperstimulation occurred exclusively in the dinoprostone group (3.0% versus 0%; *p* = 0.029). Considering that maternal factors and fetal intrauterine conditions would affect neonatal outcomes, Diguisto et al. [9] observed no difference in the rate of cesarean delivery due to nonreassuring fetal status for prolonged pregnancies between DBC group and dinoprostone group (5.8% vs. 5.3%, 95% CI 2.1–3.1%, *p* = 0.70). Jorge et al. [11] comparatively studied the safety of dinoprostone and DBC for labor induction in women at term with fetal growth restriction and no differences were observed between the two groups in terms of meconium, intrapartum fever, pH, Apgar scores or the rate of neonatal admissions. In our trial, there were totally14 cesarean section cases in dinoprostone group, including 11 cases due to fetal distress, 2 case due to failed induction, and 1 case on maternal request, accounting for 78.6% (11/14), 14.3%(2/14), 7.1%(1/14), respectively. Fetal distress caused by overstimulation of the uterus accounted for 54.5% (6/11), with the remaining 45.5% (5/11) caused by umbilical cord and placenta factors in the dinoprostone group. In contrast, there were 6 cases of cesarean section in the DBC group, 4 of which were due to fetal distress caused by umbilical cord factors, and none of the participants experienced uterine overstimulation. It was found that uterine hyperstimulation combined with abnormal FHR occurred exclusively in dinoprostone group, and DBC reduced the risk of amniotic fluid fecal staining. There was a downward trend of fetal distress in the DBC group as compared with the dinoprostone group, although the difference did not reach statistical significance (4.2% vs. 10.3%, *p* = 0.101).

As for the risk of infection, a 2015 meta-analysis [25] showed that Foley catheters for induction of labor was not associated with an increased risk of infection since patients who underwent cervical ripening using a Foley catheter had similar rates of chorioamnionitis (RR 0.96, 95% CI 0.66–1.38), endometritis (RR 1.03, 95% CI 0.66–1.6),pooled maternal infection (RR 0.95, 95% CI 0.81–1.12), and neonatal infection (RR 0.9, 95% CI 0.58–1.39) compared with those using prostaglandins. However, our research found that the rate of prenatal fever in DBC group was lower than that in dinoprostone group. The reason may be that dinoprostone is a PGE2 analog as well as potent systemic mediator of inflammation and infection, which leads to pyrexia among people during labor.

To our knowledge, this study is the first retrospective cohort study comparing the efficacy and safety of double balloon catheter (DBC) and dinoprostone and as labor-inducing agents for multipara at term. However, there are some limitations in this study. First, this study is a retrospective and single center study, the selection of labor induction method may be subjective, and the results are not so objective as the multi-center prospective studies. Second, the sample size of this study is relatively small, the rate of maternal complications such as postpartum hemorrhage, birth canal injury and the rate of neonatal complications such as neonatal asphyxia and NICU admission were very low, so it is uncertain whether there is a difference in the rate of serious complications under the two labor induction methods.

## Conclusion

Double balloon catheter and dinoprostone seem to be equally effective, but double balloon catheter seem to be more safe than dinoprostone for induction of labor for multipara at term.

## Data Availability

Access to the qualitative data will be given upon request to the corresponding author after taking any necessary precautions to safeguard participants’ privacy and confidentiality.
